# Perfect Spin Filter in a Tailored Zigzag Graphene Nanoribbon

**DOI:** 10.1186/s11671-017-2132-7

**Published:** 2017-05-18

**Authors:** Dawei Kang, Bowen Wang, Caijuan Xia, Haisheng Li

**Affiliations:** 1 School of Physics and Engineering, Henan University of Science and Technology, Luoyang, 471023 China; 2 School of Science, Xi’an Polytechnic University, Xi’an, 710048 China

## Abstract

Zigzag graphene nanoribbons (ZGNRs) are expected to serve as the promising component in the all-carbon spintronic device. It remains challenging to fabricate a device based on ZGNRs with high spin-filter efficiency and low experimental complexity. Using density functional theory combined with nonequilibrium Green’s function technique, we studied the spin-dependent transport properties of the tailored zigzag graphene nanoribbon. A perfect spin-filtering effect is found in the tailored structure of ZGNR. The nearly 100% spin-polarized current and high magneto-resistance ratio can be obtained by applying a homogeneous magnetic field across the device. The distribution of spin up and spin down states at the bridge carbon atom plays a dominant role in the perfect spin filtering. The tailoring of ZGNR provides a new effective approach to graphene-based spintronics.

## Background

Graphene has attracted much attention since it was discovered experimentally in 2004 [1]. Among all its exceptional properties, the long spin diffusion length and spin relaxation time [2] due to the low intrinsic spin-orbit and hyperfine couplings [3] are most suitable for spintronics [4], which aims at generating, controlling, and detecting spin-polarized current. Especially, zigzag graphene nanoribbons (ZGNRs) are expected to host spin-polarized electronic edge states and can serve as the promising graphene-based spintronic device. The ZGNRs are predicted to have a magnetic insulating ground state with ferromagnetic ordering at each edge and antiparallel spin orientation between two edges [5]. The graphene nanoribbons can be fabricated by cutting graphene [6], patterning epitaxially grown graphene [7] or unzipping carbon nanotubes [8, 9]. Quite recently, ZGNRs with narrow width and atomically precise zigzag edges are synthesized by a bottom-up fashion and the spin-polarized edge states are directly observed by using scanning tunneling spectroscopy [10]. A variety of spintronic devices based on GNRs with edge hydrogenations [11], nanopore [12] and different connection methods [13] have been devised. The graphene nanoscale junctions formed by two GNRs leads with different widths [14, 15] or shape [16] are found to create a spin-polarized current. A trigonal graphene [17], biphenyl molecule [18], carbon atomic chains [19], single-molecule magnet Fe_4_ [20] are inserted between two GNRs to act as a spin filter. Zeng et al. studied the spin polarized current drove by source-drain voltage in ZGNRs and found that the device can behave as a bipolar spin diode [21] or spin logic gate [22]. However, devising devices based on ZGNRs with high spin-filter efficiency and a low experimental complexity still remains a challenge.

Recent progresses in graphene engineering have made it feasible to tailor graphene into desired shapes to form a device. This method avoids the difficulties of assembling nanoscale components in the realization of complete integrated circuits. The engineering of graphene with nanometer precision has been successfully realized by scanning tunneling microscope lithography [23] and electron beam etching [24]. Actually single carbon atom can be knocked off by focused electron beam of 1 Å diameter [25]. These progresses in graphene engineering make it feasible to tailor graphene with atomic resolution into desired shape studied in this paper.

In the present work, we explore spin-dependent electron transport in a tailored zigzag graphene nanoribbon. A perfect spin-filtering effect, which only one type of spin is allowed to transport while the other type is fully blocked, is found in this structure. The designed structure can be realized by removing several carbon atoms in the selected region of zigzag graphene nanoribbon. The tailoring of GNRs to obtain a pure-spin polarized current provides a new approach to graphene-based spintronics.

## Methods

The spin-dependent transport and geometry relaxation calculation are performed by the Atomistix ToolKit [26, 27] version 2016.2 which combines density functional theory with nonequilibrium Green’s function technique. The double zeta-polarized basis sets are used for carbon and hydrogen atoms. The exchange correlation potential is described by the generalized gradient approximation with the Perdew-Burke-Ernzerhof parametrization. The density mesh cut-off is set to 150 Ry and the k-point sampling is set as 1 × 1 × 100. Geometry optimization is performed until the force becomes less than 0.01 eV/Å.

**Fig. 1:**
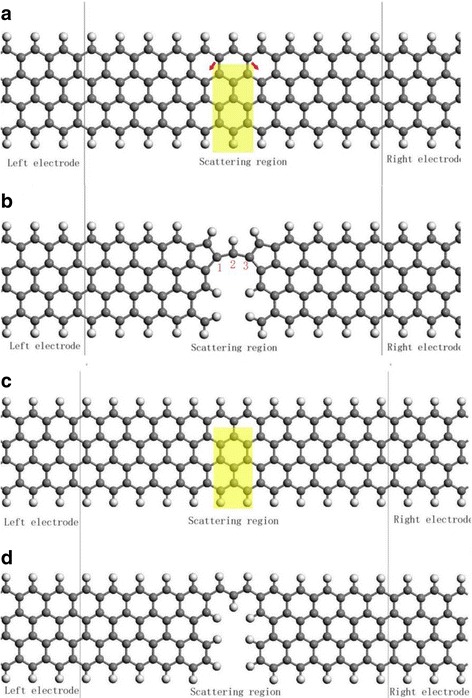
The schematic zigzag graphene device model by removing the carbon atoms in the yellow rectangular region. **b** is the relaxed structure with the dangling bonds saturated by hydrogen atoms according to **a**, **d** is the relaxed structure with the dangling bonds saturated by hydrogen atoms according to **c**

**Fig. 2:**
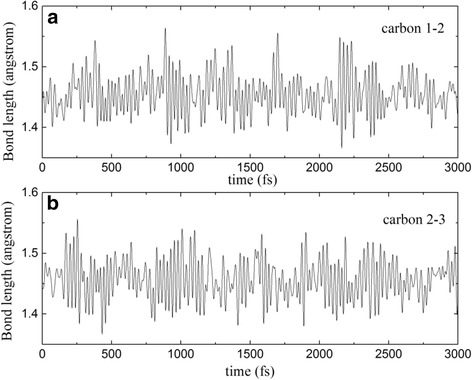
The variation of bond length in molecular dynamic simulation at 300 K. **a** The bond between carbon 1 and 2. **b** The bond between carbon 2 and 3. The carbon 1, 2, and 3 are marked with *red color* in Fig. 1 **b**

The device model studied is shown in Fig. 1. The device is divided into three regions: left electrode, scattering region and right electrode. The 4-ZGNR, which has four zigzag carbon chains across the width of the ribbon, is tailored as shown in Fig. 1a, c. Here, we consider two kinds of tailoring by removing atoms in the designated rectangular region. In the scattering region as shown in Fig. 1a, c the atoms under the yellow rectangular masks with the same area are removed by, for example, electron beams. The fully relaxed structure after these atoms being removed is shown in Fig. 1b, d correspondingly. The two carbon atoms at the tails of the red arrows in Fig. 1a will move over the arrows’ directions forming two pentagons after the atoms are removed as shown in Fig. 1b. The structure in Fig. 1c will keep its shape after the removing of atoms except some minor positional adaption as shown in Fig. 1d. The dangling bonds on the edge carbon atoms are all saturated by hydrogen atoms in order to retain the sp^2^ hybridization of carbon atoms. Hereafter we label the relaxed structure in Fig. 1b as S1 and the relaxed structure in Fig. 1d as S2. In order to test the stability of the tailored structure, we performed the ab initio molecular dynamics (MD) simulations (3 ps, with 1 fs/step) in the canonical ensemble using the VASP code [28, 29], which is verified as a powerful method to find ground state in our previous works [30, 31]. The length variation of the two bonds which connect the bridge carbon atom labeled as 2 in Fig. 1b is depicted in Fig. 2. The amplitude of the oscillation of the two bonds is about 0.1 angstrom. After running 3000 steps, the geometry is still kept, suggesting the tailored structure is stable.

The spin-dependent transmission coefficient can be calculated by

1 $$ {T}_{\sigma}\left( E, V\right)= T r\left[{\varGamma}_L{G}_{\sigma}(E){\varGamma}_R{G}_{\sigma}^{+}(E)\right], $$

where *σ* is the spin index. *G*(*E*)and *G* ^+^(*E*)are retarded and advanced Green’s function of the scattering region. *Γ*_*L*/*R*_ denotes the coupling matrix between the scattering region and the left/right electrode. The spin-dependent current through the system can be calculated with the Landauer–Buttiker formula

$$ {I}_{\sigma}(V)=\frac{e}{h}{\displaystyle \int {T}_{\sigma}}(E)\left[ f\left( E-{\mu}_L\right)- f\left( E-{\mu}_R\right)\right] d E, $$

where *f*(*E* − *μ*_*L*/*R*_) = {1 + exp [(*E* − *μ*_*L*/*R*_)/*k*_*B*_ *T*]}^−1^ is the Fermi distribution function. *μ*_*L*/*R*_ is the chemical potential of the left (right) electrode, whose value depends on the applied bias.

## Results and discussion

The magnetic edges of ZGNR electrodes have an anti-ferromagnetic coupling if no magnetic field is applied, which leads to an anti-ferromagnetic ground state. The magnetization at the two edges can be turned into the same direction by applying a magnetic field. Here, we consider two magnetic configurations of the leads which can be realized easily in experiments. The [0,0] magnetic configuration means that the two ZGNR electrodes both are anti-ferromagnetic and in ground state if there is no applied magnetic field. The [1,1] magnetic configuration means that the magnetization of the two ZGNR electrodes are parallel by applying a homogeneous magnetic field across the device [32, 33].

**Fig. 3:**
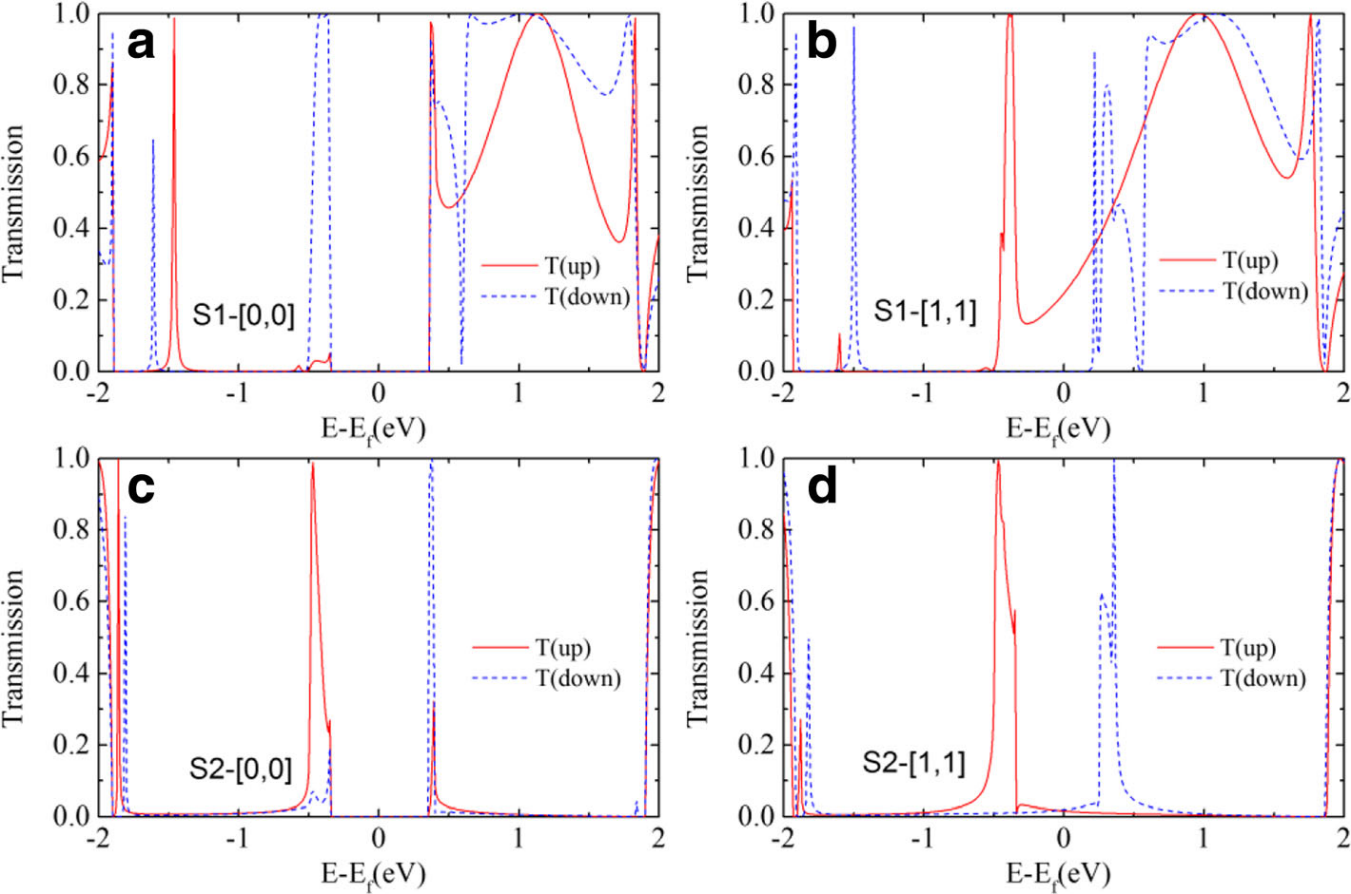
The spin-dependent transmission at zero applied bias. **a** and **b** are for structure S1; **c** and **d** are for structure S2; **a** and **c** are at magnetic configuration [0,0]; **b** and **d** are at magnetic configuration [1,1]

The spin dependent transmission coefficient is shown in Fig. 3. For both the structures S1 and S2, under the magnetic configuration [0,0] there is a transmission gap of about 0.8 eV wide which corresponds to the band gap of the ZGNR at the anti-ferromagnetic ground state, which is shown in Fig. 3a, c. As the conductance is determined by the transmission around the Fermi level, the device will conduct neither spin up current nor spin down current at low applied bias. The conduction properties of spin up and down electrons will have more differences at [1,1] magnetic configuration. As shown in Fig. 3b, the transmission for structure S1at the Fermi level has a finite value for spin up state, but it is almost equal to zero for spin down state. Here, we define the magneto-resistance ratio as (*I*_[1,1]_ − *I*_[0.0]_)/*I*_[1,1]_, where *I*_[1,1]_ and *I*_[0,0]_ is the sum of both spin up and down currents. The magneto-resistance ratio can be calculated as 100% through the equation because of the zero current at [0.0] configuration. Apart from the Fermi level, the transmission of spin down state is also blocked, but the transmission of spin up state remains finite for a wide energy range. This huge difference of the transmission for spin up and down states around Fermi level indicates that the structure S1 possesses the potential to be a perfect spin filter. As for structure S2 at magnetic configuration [1,1], which is shown in Fig. 3d, the transmission of spin up and down states also have different behaviors, but both have finite values around Fermi level. The structure S2 has the potential of spin filter to some extent, but it is not a perfect filter as the structure S1. Although the structure S2 is obtained by the removing of atoms under the same mask area, the conductance shows strong difference with structure S1 and no longer possesses the perfect spin-filtering effect.

**Fig. 4:**
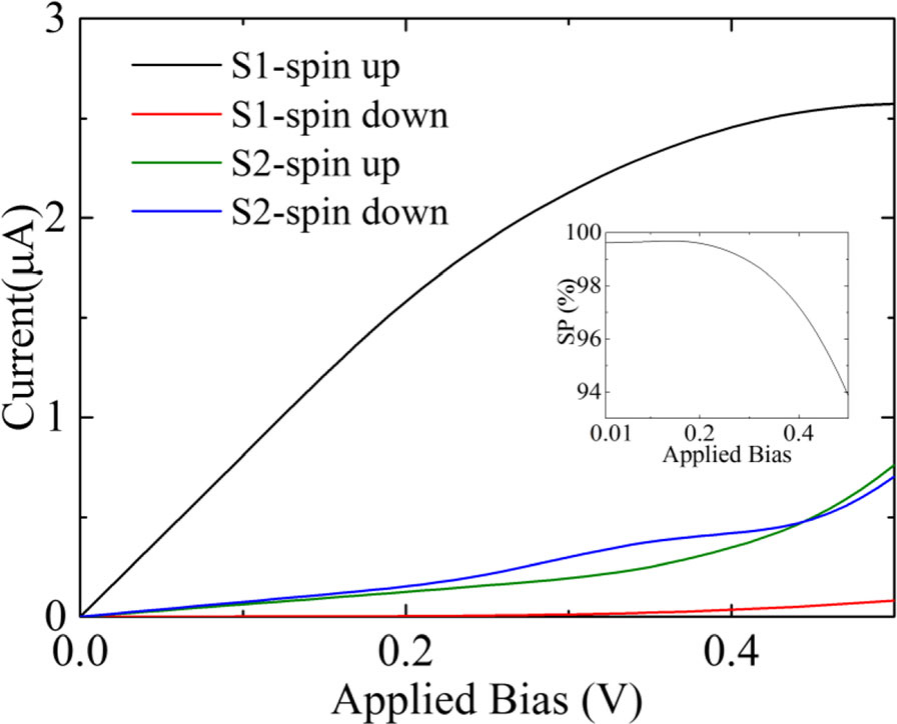
The spin-resolved current voltage characteristics of the device at [1,1] configuration. The *inset* shows the spin polarization of structure S1

In order to explore the performance of the device at finite applied bias. Figure 4 shows the calculated I-V curve of the structure S1 and the structure S2 at magnetic configuration [1,1]. As can be seen, the spin up current of the structure S1 will increase linearly with applied bias while the spin down current is nearly blocked for applied bias lower than 0.2 V. The spin polarization of the current calculated as *SP* = (*I*_↑_ − *I*_↓_)/(*I*_↑_ + *I*_↓_) reaches nearly 100% as shown in the inset of Fig. 4. The spin resolved currents of the structure S2 both increase with the applied bias and show lower spin polarization compared with structure S1. The unique structure of S1 is favorable for a nearly 100% spin polarization current. One possible application of the structure S1 may be controlling the spin current by an applied magnetic field and we can also obtain a high magneto-resistance ratio. Without magnetic field, neither spin up nor spin down current are transmitted. Applying a magnetic field will generate a nearly 100% spin polarized current.

**Fig. 5:**
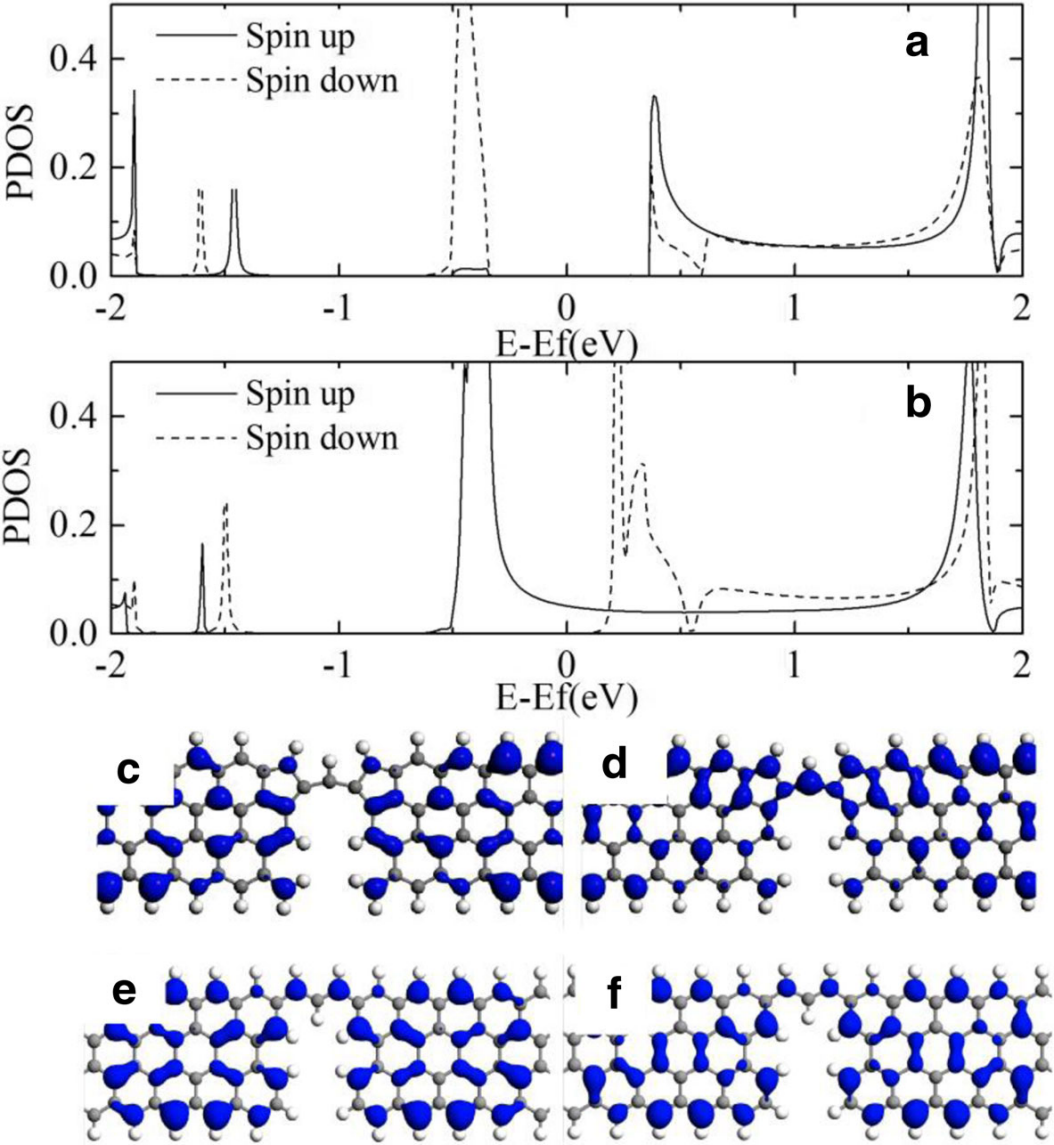
The projected density of states on the bridge carbon atom of structure S1 at **a** [0,0] and **b** [1,1] configuration. **c** and **d** show the space resolved local density of states (LDOS) for spin down and spin up at the Fermi level at [1,1] configuration. **e** and **f** show the LDOS of structure S2 for comparison

The physical reasons under the high spin polarization of structure S1 is analyzed as follows. A single carbon atom bridges the two carbon pentagons in structure S1. The scattering state distribution at this bridge carbon atom dominates the transport properties of the device. As shown in Fig. 5b, the projected density of states (PDOS) of the bridge carbon atom is depicted. The PDOS of spin up and spin down states is quite similar with the transmission coefficient shown in Fig. 3b. The PDOS of spin down state is nearly zero around the Fermi level, and the absence of PDOS of spin down state at the bridge atom brings about the discontinuity of the pathway of the spin down electron. But the PDOS of spin up state has a relatively larger value, and the spin up pathway of electron will not be broken. The different behaviors of spin up and down PDOS at the bridge atom result in the differences in spin up and down transmission coefficient. This is true for the magnetic configuration [0,0] when comparing the transmission in Fig. 3a and the PDOS in Fig. 5a. The relation of the transmission with the structure can be understood through the space resolved LDOS at Fermi level. For structure S1 at [1,1] configuration as shown in Fig. 5c, d, the spin up state has a continuous distribution on the single bridge carbon atom, but the spin down state has no distribution on it. In other words, the different coupling of spin up and down states between bridging carbon atom and carbon pentagons play an important role. Extended *π*-orbitals can form between the bridge carbon atom and the carbon pentagons for spin up state, but there is no extended *π*-orbitals for spin down state. As a result, the spin down channel will be blocked and the spin up channel is permitted at the Fermi level. But for structure S2 at [1,1] configuration as shown in Fig. 5e, f the spin up and spin down state has the similar discontinuous distribution on the three bridging carbon atoms. As a result, the spin up and down channels are all suppressed with low transmission values as shown in Fig. 3d.

**Fig. 6:**
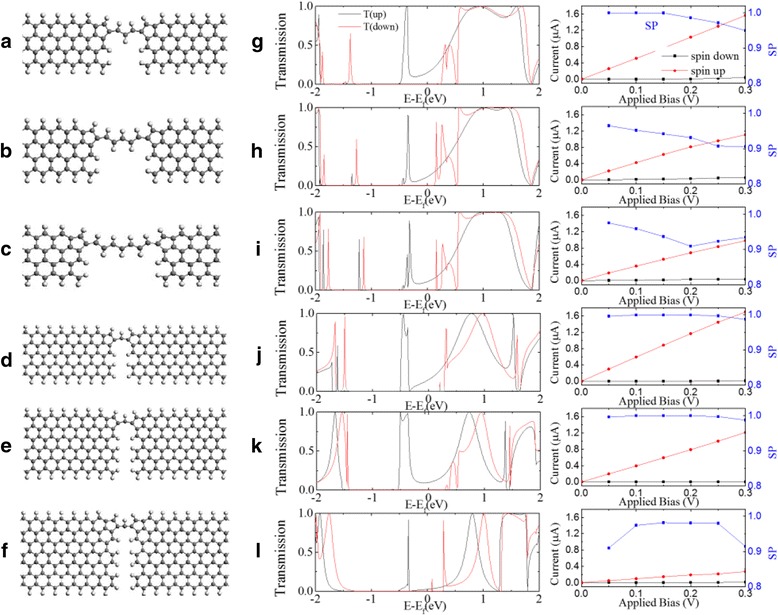
The spin-dependent transmission, spin-resolved current and spin polarization (**g**–**l**) corresponding to different tailored ZGNR structures (**a**–**f**) at magnetic configuration [1,1]. The structure **a**–**c** is optimized after symmetrically removing more carbon atoms of 4-ZGNR. The structures **d**–**f** are optimized after removing carbon atoms of 5-ZGNR, 6-ZGNR and 7-ZGNR

We also checked 4-ZGNR by removing more carbon atoms and ZGNR of different widths by removing the carbon atoms to form the pentagon structures as shown in Fig. 6. The fully relaxed structures after removing carbon atoms is shown in the left six panels of Fig. 6 and the corresponding spin-dependent transmission, current voltage characteristics, and spin polarization are shown in the right six panels. For 4-ZGNR being removed more carbon atoms, the remaining carbon line connecting the two pentagons becomes longer. With careful check of the spin dependent transmission at the Fermi level, the spin up transmission stays at a finite value but spin down transmission always stays at a nearly zero value. The perfect spin filter effect is stable. But, the turn on point to prominent value for spin down state at positive energy may become a little near the Fermi level. This is a disadvantage for obtaining high current spin polarization at low applied bias. The spin polarization as shown in Fig. 6g–i will drop to lower value with the remaining carbon lines becoming longer. For ZGNRs with different width varying from 5 to 7 the perfect spin filter effect is also retained but the spin up transmission may become very low, especially for 7-ZGNR. The Fermi level locates at the overlapping tails the two broad transmission peaks which contribute for the finite transmission at Fermi level. With the width of the ZGNR increasing the two broad peaks become narrower because of the coupling changes between bridge carbon atom and the ZGNR electrode. So, the spin up transmission drops to lower value with width changes. The spin resolved current will drop obviously as shown in Fig. 6j–l. with the increasing width of ZGNR. There are also some transmission peaks appearing near the Fermi level for spin down state with increasing the width of ZGNR. These peaks can lower the current spin polarization at low applied bias as shown in Fig. 6l. Overall, the spin-dependent transmissions always exhibit a perfect spin filtering effect at the Fermi level. So, the perfect spin filtering effect found in the tailored ZGNR structure is a steady property with proper tailoring.

## Conclusion﻿s﻿﻿

In summary, we studied the spin dependent transport in the tailored ZGNR devices. The perfect spin filtering is found in structure S1, which has a bridging carbon atom between two carbon pentagons. The spin-polarized current can be controlled by an applied magnetic field. The underlying physical origin is also explained by the LDOS distribution on the bridging carbon atom. The perfect spin filter effect is steady for tailoring with different lengths and widths. The tailoring of ZGNR to obtain the desired spin polarized current provides a new way to graphene-based spintronics.

## References

[1] Novoselov KS, Geim AK, Morozov SV, Jiang D, Zhang Y, Dubonos SV, Grigorieva IV, Firsov AA (2004). Electric Field Effect in Atomically Thin Carbon Films. Science.

[2] Tombros N, Jozsa C, Popinciuc M, Jonkman HT, van Wees BJ (2007). Electronic spin transport and spin precession in single graphene layers at room temperature. Nature.

[3] Huertas-Hernando D, Guinea F, Brataas A (2006). Spin-orbit coupling in curved graphene, fullerenes, nanotubes, and nanotube caps. Phys Rev B.

[4] Žutić I, Fabian J, Das Sarma S (2004). Spintronics: Fundamentals and applications. Rev Mod Phys.

[5] Son YW, Cohen ML, Louie SG (2006). Energy gaps in graphene nanoribbons. Phys Rev Lett.

[6] Hiura H (2004). Tailoring graphite layers by scanning tunneling microscopy. Appl Surf Sci.

[7] Berger C, Song Z, Li X, Wu X, Brown N, Naud C, Mayou D, Li T, Hass J, Marchenkov AN, Conrad EH, First PN, de Heer WA (2006). Electronic Confinement and Coherence in Patterned Epitaxial Graphene. Science.

[8] Jiao L, Zhang L, Wang X, Diankov G, Dai H (2009). Narrow graphene nanoribbons from carbon nanotubes. Nature.

[9] Kosynkin DV, Higginbotham AL, Sinitskii A, Lomeda JR, Dimiev A, Price BK, Tour JM (2009). Longitudinal unzipping of carbon nanotubes to form graphene nanoribbons. Nature.

[10] Ruffieux P, Wang S, Yang B, Sanchez-Sanchez C, Liu J, Dienel T, Talirz L, Shinde P, Pignedoli CA, Passerone D, Dumslaff T, Feng X, Mullen K, Fasel R (2016). On-surface synthesis of graphene nanoribbons with zigzag edge topology. Nature.

[11] Deng XQ, Zhang ZH, Tang GP, Fan ZQ, Yang CH (2014). Spin filter effects in zigzag-edge graphene nanoribbons with symmetric and asymmetric edge hydrogenations. Carbon.

[12] Deng XQ, Zhang ZH, Tang GP, Fan ZQ, Yang CH, Sun L (2015). The design of bipolar spin semiconductor based on zigzag–edge graphene nanoribbons. Carbon.

[13] Deng XQ, Zhang ZH, Sun L, Wu LJ (2017). Modulation of the magnetic properties in zigzag-edge graphene nanoribbons by connection sites. Org Electron.

[14] Guo J, Ouyang Y (2009). Spin-polarized edge and transport in graphene nanoscale junctions. Appl Phys Lett.

[15] Saffarzadeh A, Farghadan R (2011). A spin-filter device based on armchair graphene nanoribbons. Appl Phys Lett.

[16] Ma Z, Sheng W (2011). A spin-valve device based on dumbbell-shaped graphene nanoislands. Appl Phys Lett.

[17] Li J, Zhang ZH, Deng XQ, Fan ZQ, Tang GP (2015). Magnetic transport properties of a trigonal graphene sandwiched between graphene nanoribbon electrodes. Carbon.

[18] Wang B, Li J, Yu Y, Wei Y, Wang J, Guo H (2016). Giant tunnel magneto-resistance in graphene based molecular tunneling junction. Nanoscale.

[19] Zeng MG, Shen L, Cai YQ, Sha ZD, Feng YP (2010). Perfect spin-filter and spin-valve in carbon atomic chains. Appl Phys Lett.

[20] Zu F, Gao G, Fu H, Xiong L, Zhu S, Peng L, Yao K (2015). Efficient spin filter and spin valve in a single-molecule magnet Fe4 between two graphene electrodes. Appl Phys Lett.

[21] Zeng M, Shen L, Zhou M, Zhang C, Feng Y (2011). Graphene-based bipolar spin diode and spin transistor: Rectification and amplification of spin-polarized current. Phys Rev B.

[22] Zeng M, Shen L, Su H, Zhang C, Feng Y (2011). Graphene-based spin logic gates. Appl Phys Lett.

[23] Tapaszto L, Dobrik G, Lambin P, Biro LP (2008). Tailoring the atomic structure of graphene nanoribbons by scanning tunnelling microscope lithography. Nat Nano.

[24] Sommer B, Sonntag J, Ganczarczyk A, Braam D, Prinz G, Lorke A, Geller M (2015). Electron-beam induced nano-etching of suspended graphene. Sci Rep.

[25] Rodriguez-Manzo JA, Banhart F (2009). Creation of Individual Vacancies in Carbon Nanotubes by Using an Electron Beam of 1 Å Diameter. Nano Lett.

[26] Brandbyge M, Mozos J-L, Ordejón P, Taylor J, Stokbro K (2002). Density-functional method for nonequilibrium electron transport. Phys Rev B.

[27] José MS, Emilio A, Julian DG, Alberto G, Javier J, Pablo O, Daniel S-P (2002). The SIESTA method for ab initio order- N materials simulation. J Phys Condens Matter.

[28] Kresse G, Furthmüller J (1996). Efficient iterative schemes for ab initio total-energy calculations using a plane-wave basis set. Physical Review B.

[29] Kresse G, Furthmüller J (1996). Efficiency of ab-initio total energy calculations for metals and semiconductors using a plane-wave basis set. Comput Mater Sci.

[30] Li H, Du H, Chen W, Shan QQ, Sun Q, Guo ZX, Jia Y (2012). Threadlike Tin Clusters with High Thermal Stability Based on Fundamental Units. J Phys Chem C.

[31] Li H, Ji Y, Wang F, Li SF, Sun Q, Jia Y (2011). Ab initio study of larger Pbn clusters stabilized by Pb7 units possessing significant covalent bonding. Physical Review B.

[32] Hayakawa R, Karimi MA, Wolf J, Huhn T, Zöllner MS, Herrmann C, Scheer E (2016). Large Magnetoresistance in Single-Radical Molecular Junctions. Nano Lett.

[33] Shen H, Cresti A, Escoffier W, Shi Y, Wang X, Raquet B (2016). Peculiar Magnetotransport Features of Ultranarrow Graphene Nanoribbons under High Magnetic Field. ACS Nano.

